# Cycles of disasters in Afghanistan: The urgent call for global solidarity

**DOI:** 10.1371/journal.pgph.0002751

**Published:** 2024-01-08

**Authors:** Mohammad Yasir Essar, Sharifullah Wahdati, Brianne O’Sullivan, Arash Nemat, Karl Blanchet

**Affiliations:** 1 Department of Global Health, McMaster University, Hamilton, Ontario, Canada; 2 Afghanistan National Charity Organization for Special Diseases, Kabul, Afghanistan; 3 Temerty Faculty of Medicine, Department of Nutritional Sciences, University of Toronto, Ontario, Canada; 4 Afghanistan Institute of Nutrition and Home Economics, Kabul, Afghanistan; 5 Office of International Affairs for the Health Portfolio, Multilateral Relations Division, Public Health Agency of Canada, Ottawa, Ontario, Canada; 6 Department of Microbiology, Kabul University of Medical Sciences, Kabul, Afghanistan; 7 Department of Global Public Health, Karolinska Institutet, Solna, Sweden; 8 Geneva Centre of Humanitarian Studies, University of Geneva, Geneva, Switzerland; PLOS: Public Library of Science, UNITED STATES

On October 7, 2023, an earthquake with a magnitude of 6.3 struck Afghanistan’s western province of Herat, causing massive casualties and significant losses [1]. Initial search and rescue efforts were led by locals with support from the government of Afghanistan and non-governmental organizations (NGOs). According to early reports, at least 1482 people have died and more than 2,000 were injured, including women and children [2].

Surrounded by mountains, Afghanistan has a history of frequent natural disasters, including strong earthquakes, flash floods, and drought. In June 2022, more than 1000 people died and 1500 were seriously injured due to a strong earthquake that hit the southeastern region of the country [3]. Frequent flash floods during the months of July and August 2022 affected the lives and livelihoods of people in a number of provinces in the central, southern and south-eastern regions, approximately 16,000 families were impacted with a death toll of 192, over 11,000 houses were damaged further escalating internal displacement of people [4].

The remnants of the recent earthquakes and flash floods are compounded by a humanitarian crisis in the country, where the Afghan people were already enduring economic hardship and political instability. Since the withdrawal of the United States (US) mission and several international organizations following the August 2021 transition of power, the situation in the country has worsened significantly [5]. Restriction on women’s movement and an increased shortage of female health-care providers post August 2021 has affected access and coverage of essential health services, especially for women and children [6]. As of November 30, 2023, women and girls are not allowed to attend schools and universities. This enforcement goes against human rights and will have a negative impact on the social and economic sectors of the country in the long term. There is an urgent need for global solidarity in support of Afghan girls to ensure their voices are not silenced. Additionally, universities worldwide should establish remote and in-person educational programs for Afghan girls. This will help them pursue their dreams and become agents of social change in the future.

Afghanistan remains one of the most at risk countries of worsening humanitarian crises with a staggering 28.3 million people (approximately 70% of the country’s population) currently in need of humanitarian support [7]. Poverty impacts more than 90% of the population, with 91% of the average household’s income being spent on food, pushing many families to resort to coping strategies such as rationing [8].

The country faces a dire food insecurity and malnutrition situation. As per the latest UN’s Integrated Food Security Phase Classification (IPC) 2023 analysis, 17.2 million Afghans face high levels of food insecurity and were classified in Crisis or Emergency (IPC Phase 3 or 4) in April 2023 [9]. This situation is projected to further deteriorate due to recurring natural disasters, high rates of internal and cross-border displacement from Pakistan, as well as the harsh winter season expected in the coming months. With residents of Afghanistan forced to make hard choices to access food, women and children are at particularly higher risk of hunger and related preventable diseases. Furthermore, a significant proportion of the population already suffers from malnutrition. In children under five, 900,000 suffer from severe acute malnutrition and 2.3 million are affected by moderate acute malnutrition. In addition, stunting, a chronic malnutrition indicator, has increased from 38% in 2018 to 44.7% in 2023 among children under-5 [10].

Over the last few years, Afghanistan has grappled with an endless cycle of crises and disasters, and there is little support and solidarity from the global community towards Afghanistan. With the withdrawal of the US, many NGOs halted their activities in Afghanistan due to political reasons, especially after the Taliban imposed a ban on women workers in December 2022 [11]. While the transition of the government caused turbulence, civilians, especially women’s rights groups, have not been able to function properly. The Humanitarian Response Plan 2023 for Afghanistan highlighted a total funding gap of 78% required to reach people in need of assistance in areas of health, food security, nutrition, education, shelter and protection, these funding gaps will significantly affect the ability of humanitarian organizations to deliver emergency relief and services [12]. As a result of reduced funding, 18 million people in crises levels of food insecurity did not receive food assistance, and another 3.4 million people received only half rations [12].

The healthcare system in the country is fragile and dependent on foreign aid to operate, with significant shortages in trained health care workers and essential medical supplies. Since August 2021, the UN has continuously appealed for foreign aid to support the humanitarian response in Afghanistan. According to the United Nations Development Programme (UNDP), a sustained inflow of foreign aid, of around $3.7 billion has helped prevent the collapse of government [13]. Despite this, several international NGOs have ended their support to critical services due to funding shortages. In August 2023, the International Committee of the Red Cross (ICRC) halted its services at 25 major hospitals across Afghanistan that disrupted services to a vast proportion of the population and affected the employment of as many as 10,000 health-care providers [14]. Several other organizations providing essential healthcare services face similar challenges and are on the verge of closing critical services if the funding gaps are not met.

At this critical juncture, the health of the Afghans is of utmost importance, particularly after major natural disasters and amid heightened risks of subsequent disease outbreaks. It is well documented that following such natural disasters there is an increased risk of disease spread, due to damaged health infrastructure, inadequate living conditions, and crowded settings. We therefore urge international organizations to draw their attention to the critical situation in Afghanistan. For continuation of key services in the country, a funding gap of USD 2.52 billion must be filled by NGOs, the World Bank, and the UN to ensure food security, health, and nutrition support to affected populations in 2023 [13]. The economic sector is also critical–with the poverty rate escalating. Tackling this situation requires the engagement of women in all sectors. The World Economic Forum has reported that restrictions over women’s engagement in private sector employment could lead to a further $1.5 billion losses of output by 2024 [15]. Furthermore, international NGOs should engage with the government to find means of working in the country under the right conditions–with women being engaged in all sectors. It is only then that Afghanistan can slowly move forward towards rebuilding sustainable systems that would benefit the population in long-term. In the health and nutrition sectors, where the situation is alarmingly critical, the international community can allocate immediate, lifesaving resources as well as help ensure long-term gains through building human resource capacities, investing in preventive care strategies, and leveraging community approaches for improved access and coverage of health services.
